# Low levels of hemoglobin associate with critical illness and predict disease course in patients with ANCA-associated renal vasculitis

**DOI:** 10.1038/s41598-022-23313-7

**Published:** 2022-11-04

**Authors:** Eva Baier, Desiree Tampe, Samy Hakroush, Björn Tampe

**Affiliations:** 1 Department of Nephrology and Rheumatology, University Medical Center Göttingen, Göttingen, Germany; 2 Institute of Pathology, University Medical Center, Göttingen, Germany; 3 SYNLAB Pathology Hannover, SYNLAB Holding Germany, Augsburg, Germany

**Keywords:** Kidney diseases, Risk factors, Outcomes research

## Abstract

Antineutrophil cytoplasmic antibody (ANCA)-associated vasculitis (AAV) is a small vessel vasculitis often leading to critical illness by multi-organ failure. Data for patients with specifically ANCA-associated renal vasculitis requiring intensive care unit (ICU) supportive care are limited and have mainly focused on long-term renal and overall outcome. Particularly, data on critical illness during the initial course of disease are scarce and remain poorly determined. Therefore, the purpose of this retrospective study was to identify predictors of critical illness in a cohort of patients with ANCA-associated renal vasculitis. We retrospectively included a total number of 53 cases with confirmed ANCA-associated renal vasculitis between 2015 till 2020 in a single-center cohort study. We here identified an association between low hemoglobin levels and requirement of ICU supportive care in patients with ANCA-associated renal vasculitis. Furthermore, levels of hemoglobin below 9.8 g/dL at admission independently predicted prolonged requirement of ICU supportive care in critically ill patients with ANCA-associated renal vasculitis. These findings confirm that low levels of hemoglobin negatively affect short-term outcome and could further improve our current understanding for the role of anemia in ANCA-associated renal vasculitis.

## Introduction

Antineutrophil cytoplasmic antibody (ANCA)-associated vasculitis (AAV) is a small vessel vasculitis according to the 2012 revised Chapel Hill Consensus Conference Nomenclature of Vasculitides, most frequently presenting as microscopic polyangiitis (MPA) or granulomatosis with polyangiitis (GPA)^1,2^. Pauci-immune necrotizing and crescentic ANCA glomerulonephritis (GN) and diffuse pulmonary hemorrhage are serious disease manifestations of AAV that determine prognosis and survival. ANCA-associated renal vasculitis occurs in 20–50% of AAV patients at disease onset and in 70–80% of patients during the further course of disease^2^. Renal involvement is a severe complication of AAV resulting in acute kidney injury (AKI), progression into chronic kidney disease (CKD), requirement of kidney replacement therapy (KRT), or death^2^. Therefore, prompt initiation of immunosuppressive drugs for remission induction is critical for patient outcomes. In generalized and severe forms, conventional induction treatment combines high doses of glucocorticoids and cyclophosphamide (CYC)^3^. In addition, the anti-CD20 monoclonal antibody rituximab (RTX) can be used as an alternative or in combination with CYC^4–6^. Finally, additional plasma exchange (PEX) can be considered in case of severe kidney injury and/or alveolar hemorrhage. Under these regimens, AAV remission is achieved in 60–80% of these patients^4–8^. However, some patients experience resistance to therapy or relapsing disease. Moreover, a high mortality rate is observed in AAV patients, ranging from 10 to 15% within the first year following remission induction^9,10^. Particularly, the main causes of early death being infection events and vasculitis manifestations, especially ANCA-associated renal vasculitis^9,10^. Mortality rates of up to 20% after 5 years have been observed, and mortality has been shown to be higher with MPA than with GPA^9^. To date, patients with AAV and concomitant critical illness requiring intensive care unit (ICU) treatment have not been extensively analyzed. Moreover, these studies included AAV patients with manifestations related to vasculitis activity, but also other complications. Finally, most previous studies focused on critical illness due to pulmonary hemorrhage and respiratory failure^11–19^. Data for AAV patients admitted to the ICU due to biopsy-proven ANCA-associated renal vasculitis is limited and have mainly focused on long-term renal and overall outcome^18–22^. Particularly, data on critical illness during the initial course of disease are scarce and remains poorly determined. Therefore, the purpose of this retrospective study was to identify predictors of critical illness in a previously described cohort of patients with biopsy-proven ANCA-associated renal vasculitis^23–27^.

## Results

**Figure 1:**
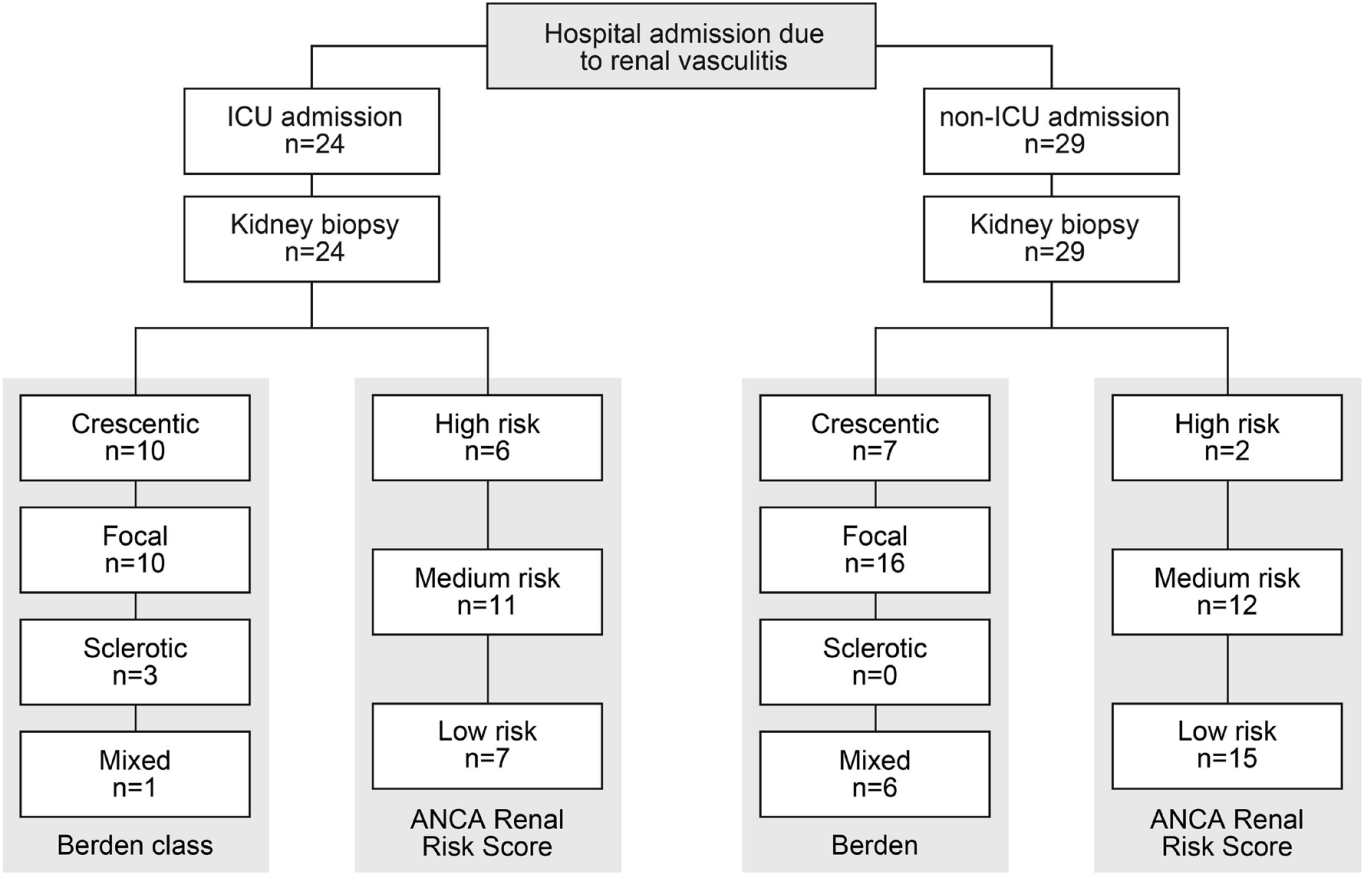
STROBE flow chart of the total patient cohort of biopsy-proven ANCA-associated renal vasculitis.

In total, 53 cases with biopsy-proven ANCA-associated renal vasculitis were included (Fig. 1). 23/53 (43.4%) were female and all were Caucasian (Table 1). The median (IQR) age at diagnosis was 65 (54.5–74.5) years, 26/53 (49.1) patients were categorized as MPA and remainder as GPA with new diagnosis of ANCA-associated renal vasculitis in the majority of cases (Table 1). As per inclusion criteria of ANCA-associated renal vasculitis, 16/53 (30.2%) required KRT within 30 days after admission, none of the patients required mechanical ventilation due to respiratory failure, and 1 patient was ventilated due to status epilepticus (Table 1). This diagnostic workup, initiation of remission induction therapy and duration until clinical improvement resulted in a median (IQR) in-hospital length of stay of 12 (8.5–19.5) days in the non-ICU group, while severe ANCA-associated renal vasculitis with critical illness and requirement of ICU supportive care prolonged the median hospitalization time to 23.5 (12.8–41.5) days. Among these 24/53 (45.3%) of patients requiring ICU supportive care, ICU length of stay (IQR) was 4 (3–8) days, and ICU mortality was 3/24 (12.5%, Table 1). Critical illness due to severe ANCA-associated renal vasculitis was confirmed by established scoring systems, the median (IQR) SAPS II was 29.5 (24.25–34.75) points, SOFA 3 (1.5–4) points, and APACHE II 13 (8–17) points (Table 1). Parameters for calculation of SAPS II, SOFA and APACHE in patients requiring ICU supportive care confirmed that kidney injury was the predominant cause for ICU admission (Table 2). All 53 patients had a diagnostic kidney biopsy, the Berden class was sclerotic in 3 patients, crescentic in 17 patients, focal in 26 patients, and mixed in 7 patients (Table 1). 44/53 (83%) of patients also had extrarenal disease (31 with lung, 7 with alveolar hemorrhage, 9 with sinus, 12 with joint, 4 with ear, 3 with eye, 6 with peripheral nerve and 9 with skin involvement, Table 1).

**Table 1:** Clinical characteristics of the total patient cohort of ANCA-associated renal vasculitis.

	Total cohort (n = 53)	ICU (n = 24)	Non-ICU (n = 29)	*p* value
Female sex—no. (%)	23 (43.4)	9 (37.5)	14 (48.3)	0.4308
Age—years	65 (54.5–74.5)	70 (55.75–76)	60 (53.5–69.5)	0.1212
**ANCA diagnosis**				
MPA—no. (%)	26 (49.1)	13 (54.2)	13 (44.8)	
GPA—no. (%)	27 (50.1)	11 (45.8)	16 (55.2)	0.4984
**ANCA subtype**				
MPO—no. (%)	26 (49.1)	12 (50)	14 (48.3)	
PR3—no. (%)	27 (50.1)	12 (50)	15 (51.7)	0.9005
Relapse—no. (%)	8 (15.1)	3 (12.5)	5 (17.2)	0.6313
BVAS—points	18 (15–20.5)	18 (15.3–19.8)	18 (14.5–21)	0.9609
KRT within 30 days—no. (%)	16 (30.2)	15 (62.5)	1 (3.4)	< 0.0001
Mechanical ventilation—no. (%)	1 (1.9)	1 (4.2)	0 (0)	0.2671
ICU length of stay—days	NA	4 (3–8)	NA	NA
ICU mortality—no. (%)	NA	3 (12.5)	NA	NA
SAPS II—points	NA	29.5 (24.3–34.8)	NA	NA
SOFA—points	NA	3 (1.5–4)	NA	NA
APACHE—points	NA	13 (8–17)	NA	NA
In-hospital length of stay—days	16 (9–27.5)	23.5 (12.75–41.5)	12 (8.5–19.5)	0.0043
Follow-up time—days	574 (276–1147)	410 (62.25–1051)	599 (436.5–1377)	0.0814
**Extrarenal manifestations**				
Total—no. (%)	44 (83)	17 (70.8)	27 (93.1)	
Lung involvement—no. (%)	31 (58.5)	16 (66.7)	15 (51.7)	0.2718
Alveolar hemorrhage—no. (%)	7 (13.2)	4 (16.7)	3 (10.3)	0.4986
Sinus involvement—no. (%)	9 (17)	2 (8.3)	7 (24.1)	0.1272
Joint involvement—no. (%)	12 (22.6)	5 (20.8)	7 (24.1)	0.7748
Ear involvement—no. (%)	4 (7.5)	0 (0)	4 (13.8)	0.0585
Eye involvement—no. (%)	3 (5.7)	1 (4.2)	2 (6.9)	0.6686
Nerve involvement—no. (%)	6 (11.3)	1 (4.2)	5 (17.2)	0.1348
Skin involvement—no. (%)	9 (17)	2 (8.3)	7 (24.1)	0.1272
**Histopathological lesions**				
Normal glomeruli—% of total	48.9 (26.2–73)	39.9 (11.3–68)	54.6 (36.7–81.7)	0.0294
Crescentic glomeruli—% of total	30.8 (9.8–55.1)	40.6 (12.7–62.4)	27.3 (5.6–48.4)	0.1189
Necrotic glomeruli—% of total	15.2 (0–44.7)	20.5 (1.9–62.5)	12.5 (0–35.3)	0.2124
Sclerotic glomeruli—% of total	5.1 (0–26.3)	8.8 (0–29.9)	5 (0–19)	0.4831
IF/TA—%	20 (10–40)	20 (10–37.5)	20 (7.5–40)	0.8902
**Berden class**				
Sclerotic class—no. (%)	3 (5.7)	3 (12.5)	0 (0)	
Crescentic class—no. (%)	17 (32.1)	10 (41.7)	7 (24.1)	
Focal class—no. (%)	26 (49.1)	10 (41.7)	16 (55.2)	
Mixed class—no. (%)	7 (13.2)	1 (4.2)	6 (20.7)	0.0443
**ANCA Renal Risk Score**				
High risk—no. (%)	8 (15.1)	6 (25)	2 (6.9)	
Intermediate risk—no. (%)	23 (43.4)	11 (45.8)	12 (41.4)	
Low risk—no. (%)	22 (41.5)	7 (29.2)	15 (51.7)	0.1043
Categorization of normal glomeruli				
Normal glomeruli 0–25%—no. (%)	12 (22.6)	10 (41.7)	2 (6.9)	
Normal glomeruli 26–50%—no. (%)	16 (30.2)	5 (20.8)	11 (37.9)	
Normal glomeruli 51–75%—no. (%)	14 (26.4)	8 (33.3)	6 (20.7)	
Normal glomeruli 76–100%—no. (%)	11 (20.8)	1 (4.2)	10 (34.5)	0.0019
**Initial treatment**				
Use of PEX—no. (%)	20 (37.7)	15 (62.5)	5 (17.2)	0.0007
Sessions of PEX—no	10 (18.9)	5 (5–7)	5 (5–5)	0.8985
Intravenous steroid pulse—no. (%)	37 (69.8)	22 (91.7)	15 (51.7)	0.0016
Oral steroids—no. (%)	53 (100)	24 (100)	29 (100)	NA
**Further remission induction**				
RTX—no. (%)	19 (35.8)	8 (33.3)	11 (37.9)	
CYC—no. (%)	25 (47.2)	12 (50)	13 (44.8)	
RTX/CYC—no. (%)	8 (15.1)	3 (12.5)	5 (17.2)	
Other—no. (%)	1 (1.9)	1 (4.2)	0 (0)	0.6694

Continuous variables are expressed as median and IQR, categorical variables are presented as frequency and percentage. For group comparisons, the Mann–Whitney *U* test was used to determine differences in medians. Non-parametric between-group-comparisons were performed with Pearson’s Chi-square test.

**Table 2:** Parameters for calculation of SAPS II, SOFA and APACHE in patients requiring ICU supportive care.

	ICU (n = 24)
Age—years	70 (55.75–76)
Heart rate—per minute	79.5 (72–87)
Systolic blood pressure—mmHg	130 (112–145)
Mean arterial pressure—mmHg	100 (93.8–108.8)
Body temperature—°C	36.7 (36–37)
GCS 14–15 points—no. (%)	23 (95.8)
GCS < 6 points—no. (%)	1 (4.2)
Not on mechanical ventilation—no. (%)	23 (95.8)
PaO_2_/FiO_2_ > 200—no. (%)	1 (4.2)
Urine output ≥ 1000 mL per day—no. (%)	17 (70.8)
Urine output 500–999 mL per day—no. (%)	6 (25)
Urine output < 500 mL per day—no. (%)	1 (4.2)
Serum creatinine—mg/dL	3.1 (1.6–5.7)
Blood urea nitrogen—mg/dL	58.5 (36.8–83.8)
Sodium—mmol/L	138 (135.3–140)
Potassium—mmol/L	4.4 (4.2–4.7)
Bilirubin—mg/dL	0.4 (0.3–0.775)
White blood cells— × 1000/µL	11.5 (9.4–14.9)
Platelets— × 1000/µL	300 (251–473)
pH	7.43 (7.35–7.47)
Hematocrit—%	27.5 (23.7–28.5)

Continuous variables are expressed as median and IQR, categorical variables are presented as frequency and percentage.

Comparison of the ICU and the non-ICU groups are detailed in Table 1. Groups were similar regarding gender, age, AAV subtype and relapsing disease. Disease activity assessed by BVAS showed no statistical difference between groups. Risk of severe kidney injury reflected by requirement of KRT during the further disease course within 30 days after admission was significantly increased in the ICU as compared to the non-ICU group. In addition, in-hospital length of stay was significantly longer in patients requiring ICU supportive care. Interestingly, a significant lower fraction of normal glomeruli was present in the ICU group that was not attributed to any other histopathological lesion typical for ANCA-associated renal vasculitis. While distribution of Berden class ANCA-associated renal vasculitis differed between groups, ARRS was comparable between the ICU and the non-ICU groups. Finally, extrarenal AAV manifestations were comparable in both groups. With regard of AAV treatment, the use of PEX and pulse steroids were significantly more in the ICU group, while further remission induction regimens did not differ between the ICU and the non-ICU groups. In summary, severe kidney injury was the main reason for ICU admission in this cohort of biopsy-proven ANCA-associated renal vasculitis and associated with requirement of KRT during the further course of the disease. Critical illness due to renal failure was associated with increased PEX and intravenous steroid pulse treatments in this subgroup.

**Figure 2:**
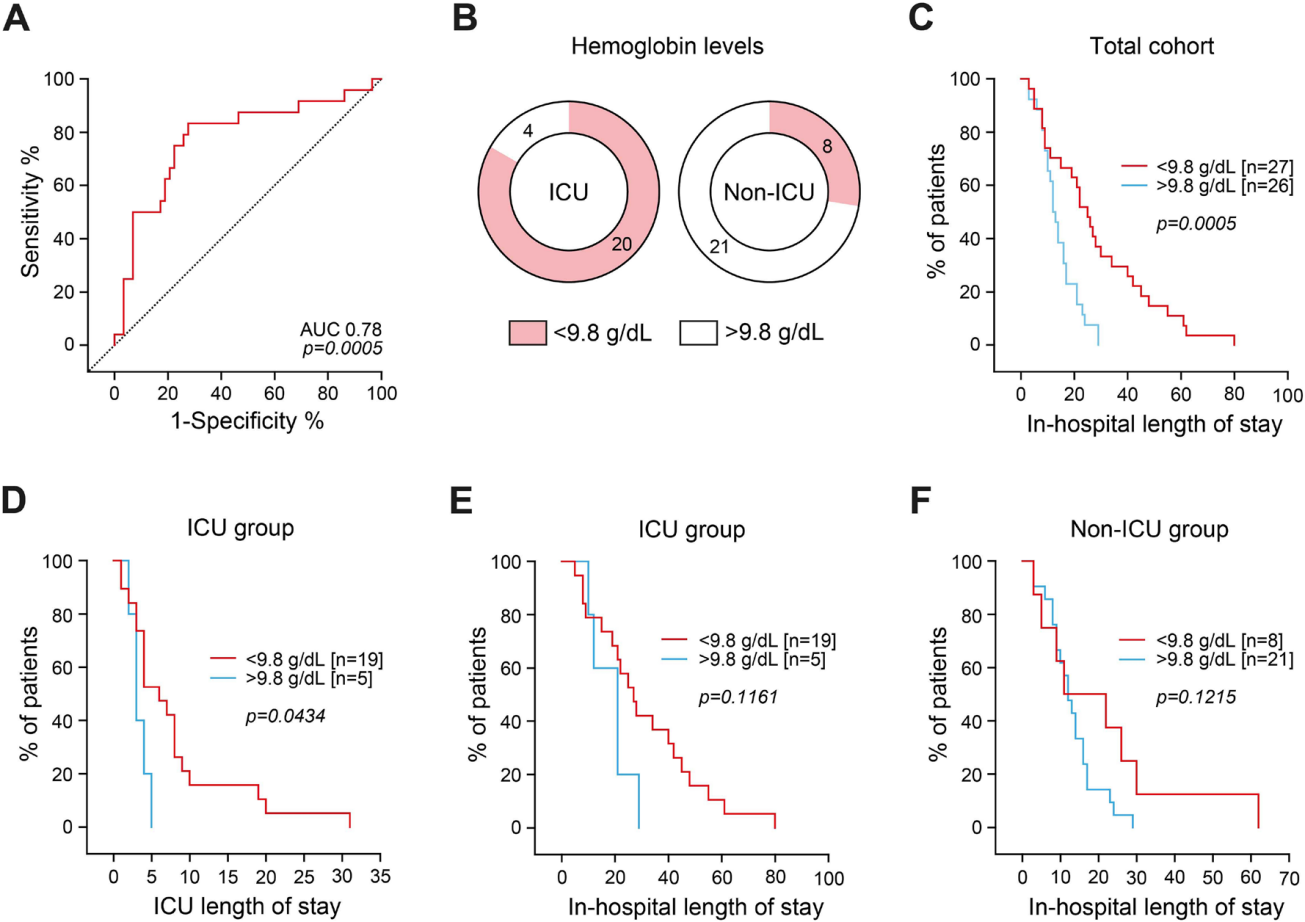
Low levels of hemoglobin predict disease course in critically ill patients with biopsy-proven ANCA-associated renal vasculitis. (**A**) Cutoff points for hemoglobin levels on the ROC that maximized Youden’s index identified hemoglobin levels < 9.8 g/dL to be significantly associated with ICU admission (sensitivity: 83.3%, specificity: 72.4%, *p* = 0.0005). (**B**) Frequency after group separation for hemoglobin levels at 9.8 g/dL in the ICU as compared to the non-ICU group. (**C**–**F**) Survival analysis of in-hospital and ICU lengths of stay after group separation for hemoglobin levels at 9.8 g/dL.

We next analyzed parameters at admission in association with requirement of ICU supportive care. Markers of kidney injury including serum creatinine levels, eGFR decline and proteinuria were more severely increased in the ICU group (Table 3). Additionally, levels of hemoglobin and hematocrit at admission were significantly lower in the ICU group (Table 3). Finally, levels of C-reactive protein (CRP) and gamma-glutamyl transferase (GGT) were significantly elevated in the ICU as compared to the non-ICU group (Table 3). Confirmed by multiple logistic regression, low levels of hemoglobin at admission were independently associated with ICU admission (*p* = 0.0198, Table 4). Interestingly, hemoglobin levels and hematocrit were comparable between the subgroups of MPO-ANCA and PR3-ANCA-associated renal vasculitis, excluding differences due to ANCA subtype in ANCA-associated renal vasculitis (Table 5). ROC analysis confirmed hemoglobin levels < 9.8 g/dL to be significantly associated with ICU admission (sensitivity: 83.3%, specificity: 72.4%, *p* = 0.0005, Fig. 2A,B). In addition, hemoglobin levels < 9.8 g/dL were associated with prolonged in-hospital length of stay in the total cohort of ANCA-associated renal vasculitis (median: 25 vs. 12.5 days, *p* = 0.0005, Fig. 2C). Interestingly, low levels of hemoglobin were also associated with specifically prolonged ICU length of stay in the ICU group (median: 6 vs. 3 days, *p* = 0.0434, Fig. 2D), but not total in-hospital length of stay in the ICU (median: 27 vs. 21 days, *p* = 0.1161, Fig. 2E) or non-ICU group (median: 16.5 vs. 12 days, *p* = 0.1215, Fig. 2F). These results confirm that low hemoglobin levels at admission associated with critical illness and requirement of ICU supportive care, but also predicted disease course specifically in this subgroup of severe ANCA-associated renal vasculitis.

**Table 3:** Parameters at admission in ICU and non-ICU patients with ANCA-associated renal vasculitis.

Laboratory parameters at admission	ICU (n = 24)	Non-ICU (n = 29)	*p* value
Serum creatinine—mg/dL	3.145 (1.555–5.655)	1.49 (0.93–3.16)	0.0268
eGFR—mL/min/1.73 m^2^	16.75 (8.575–32.55)	37.7 (15.65–92.75)	0.0140
Potassium—mmol/L	4.4 (4.2–4.7)	4.2 (4–4.5)	0.1246
uPCR—mg/g creatinine	1447 (656.5–2866)	729.1 (456.2–1377)	0.0154
uACR—mg/g creatinine	687.8 (223.9–1190)	416.3 (109.5–768.1)	0.1620
Hemoglobin—g/dL	8.9 (7.7–9.5)	10.6 (9.55–12.85)	< 0.0001
Hematocrit—%	27.5 (23.7–28.5)	31.5 (28.15–39.6)	0.0003
CRP—mg/L	73.1 (32.78–160.3)	33.4 (7.95–85.2)	0.0346
ALT—U/L	25.5 (11–43.75)	12.5 (8.25–25)	0.1429
AST—U/L	26 (18.75–34.5)	20 (15–27)	0.0706
GGT—U/L	65.5 (22.25–116.8)	30 (18.5–57.5)	0.0448
AP—U/L	85 (68.75–131.5)	90 (66–106)	0.6819
Bilirubin—mg/dL	0.4 (0.3–0.775)	0.3 (0.3–0.65)	0.1031
Albumin—g/dL	2.6 (2.1–2.9)	2.85 (2.675–3.1)	0.1468
Uric acid—mg/dL	6.9 (5.25–7.55)	6.25 (4.85–6.65)	0.2876
LDH—U/L	263 (206.5–303)	281.5 (243.5–316)	0.3824
INR—ratio	1.1 (1–1.2)	1 (1–1.2)	0.6268
aPTT—s	27 (24–32.5)	28 (26–31.25)	0.6335

Continuous variables are expressed as median and IQR, the Mann–Whitney *U* test was used for group comparisons to determine differences in medians.

**Table 4:** Multiple logistic regression of parameters associated with ICU admission. Significant values are in italics.

Parameter	β	*p* value
Serum creatinine—mg/dL	0.1707	0.3160
eGFR—mL/min/1.73 m^2^	− 0.0748	0.6580
uPCR—mg/g creatinine	0.2697	0.0690
Hemoglobin—g/dL	− 0.3502	0.0198
Hematocrit—%	1.5450	0.1503
CRP—mg/L	0.1638	0.2620
GGT—U/L	0.2179	0.1334

**Table 5:** Hemoglobin levels and hematocrit in MPO-ANCA and PR3-ANCA-associated renal vasculitis.

Laboratory parameters at admission	MPO-ANCA (n = 24)	PR3-ANCA (n = 29)	*p* value
Hemoglobin—g/dL	9.9 (8.4–11.5)	9.6 (8.9–11.4)	0.9894
Hematocrit—%	28.9 (25.8–34.4)	28.9 (27.7–35.1)	0.7541

Continuous variables are expressed as median and IQR, the Mann–Whitney *U* test was used for group comparisons to determine differences in medians.

## Discussion

Most frequent reasons for ICU admission in AAV patients are severe respiratory insufficiency due to diffuse alveolar hemorrhage, sepsis and/or pneumonia, and acute abdomen due to bowel infarction^28^. Therefore, most previous studies focused on critical illness due to pulmonary hemorrhage and respiratory failure in AAV^11–19^. Contrasting to this, data for AAV patients admitted to the ICU due to biopsy-proven ANCA-associated renal vasculitis is limited and have mainly focused on long-term renal and overall outcome^18–22^. Because we here focused on biopsy-proven ANCA-associated renal vasculitis without requirement of mechanical ventilation due to pulmonary AAV manifestation and consecutive respiratory failure, renal involvement was higher in the present study as compared to most previous reports^11–22^. In our cohort, the median (IQR) ICU length of stay was 4 (3–8) and shorter than most previous reports focusing on critical illness due to pulmonary hemorrhage and respiratory failure in AAV^14–19^. In studies focusing on ANCA-associated renal vasculitis, ICU length of stay was comparable^18–20^. We here identified an association between low hemoglobin levels and requirement of ICU supportive care in patients with ANCA-associated renal vasculitis. Particularly, low hemoglobin levels at admission associated with requirement of ICU supportive care, but also predicted disease course specifically in ANCA-associated renal vasculitis patients with critical illness requiring ICU supportive care. Anemia has already been reported as a complication in other autoimmune and inflammatory diseases^29–32^. Anemia that occurs in the context of autoimmune and inflammatory diseases is known as anemia of chronic disease (ACD)^33^. The mechanisms of ACD are thought to involve changes in iron metabolism, inadequate response of erythropoiesis, and shortening of the erythrocytes’ lifespan^33^. Complications associated with ACD include infections, malignancies, autoimmune diseases, chronic rejection after transplantation, and chronic kidney disease (CKD). ACD is an important complication in ANCA-associated renal vasculitis, and the cause of anemia in ANCA-associated renal vasculitis are expected to be multifactorial^34^.

Generally, anemia in elderly patients is associated with poor outcome such as hospitalization and mortality^35^. In patients with heart failure, anemia is associated with increased mortality^36^. Severe anemia could result in a reduction of oxygen supply, negatively affecting outcome during critical illness^37^. In addition, several studies have demonstrated that anemia is a risk factor for renal dysfunction in various kidney diseases^38,39^. While anemia has already been reported to associate with prolonged ICU or in-hospital length of stay and increased mortality among critically ill patients with sepsis, cardiogenic shock, or trauma, some other investigations did not confirm these observations^40,41^. Therefore, the impact of anemia on outcome could differ between subgroups of critically ill patients based on the underlying diseases, and the pathogenesis of anemia may impact the prognosis of patients with critical illness. Anemia has already been described in most patients with ANCA-associated renal vasculitis, with renal anemia being the predominant type^34^. Consequently, the combination of severe anemia and ANCA-associated renal vasculitis have been shown to lead to poor long-term renal prognosis and shortened lifespan^34^. While most previous studies focused on critical illness due to pulmonary hemorrhage and respiratory failure, we here focused on critically patients specifically with ANCA-associated renal vasculitis^11–19^. Moreover, the impact of anemia on long-term outcome of ANCA-associated renal vasculitis has been a matter of debate^21,22^. Our observation that low levels of hemoglobin at admission correlated with prolonged requirement of ICU supportive care in this subgroup confirms that severe anemia also affects short-term disease course in critically ill patients with biopsy-proven ANCA-associated renal vasculitis. Therefore, additional studies to validate these results may expand our current knowledge about the specific causes and consequences of anemia in ANCA-associated renal vasculitis with critical illness. Moreover, especially critically ill patients with ANCA-associated renal vasculitis may benefit from specific therapeutic interventions to adequately increase hemoglobin levels. Interestingly, there was no difference in hemoglobin levels between MPO-ANCA and PR3-ANCA-associated renal vasculitis, excluding differences due to ANCA subtype itself. We identified a significant lower fraction of normal glomeruli that was present in the ICU group and not attributed to any other histopathological lesion typical for ANCA-associated renal vasculitis. This observation requires further investigation to identify distinct histopathological lesions that associate with renal anemia in ANCA-associated renal vasculitis. Therefore, the pathogenesis and severity of anemia in patients with ANCA-associated renal vasculitis may differ from those of anemia in patients with other autoimmune inflammatory diseases.

Our study has several important strengths. First, an important characteristic of our study design was to utilize non-ICU patients with ANCA-associated renal vasculitis as the comparison group limiting a selection bias. Second, all patients had biopsy-proven ANCA-associated renal vasculitis and none required mechanical ventilation due to pulmonary AAV manifestation and consecutive respiratory failure. Third, remission induction therapy did not differ among groups and excluding a significant impact on disease course. Our study has also several limitations. First, the relatively small number of patients and its monocentric design. Second, the retrospective study design needs validation in independent and prospective cohorts. Third, we retained the inclusion to patients with severe ANCA-associated renal vasculitis confirmed by diagnostic kidney biopsy. Therefore, these results may not be generalizable to all AAV patients, especially with extrarenal manifestations and milder symptoms. Finally, treatment regimens were equally distributed in the ICU and non-ICU groups but not protocolized, and still might have influenced individual outcomes. Nevertheless, our finding that low levels of hemoglobin independently predict short-term disease course in critically ill patients with biopsy-proven ANCA-associated renal vasculitis is of relevance. Since identification of patients at risk for a more severe disease course is of relevance for treating intensivists, these findings could further improve our current understanding for the role of anemia in ANCA-associated renal vasculitis as it has already been observed in other autoimmune and inflammatory diseases. Our findings that low levels of hemoglobin negatively affects short-term outcome cohort could further improve our current understanding for the role of anemia in ANCA-associated renal vasculitis.

## Methods

### Ethics declaration

The study was conducted according to the guidelines of the Declaration of Helsinki and approved by the Institutional Review Board of the University Medical Center Göttingen, Germany (no. 4/8/19). Informed written consent was obtained from all subjects involved in the study for the use of routinely collected data for research purposes as part of their regular medical care in the contract with the University Medical Center Göttingen.

### Study population

A total number of 53 cases admitted the University Medical Center Göttingen with confirmed ANCA-associated renal vasculitis retrospectively included between 2015 till 2020, the patient cohort has in part previously been described^23–27^. None of the patients required mechanical ventilation due to respiratory failure, and 1 patient was ventilated due to status epilepticus. Medical records were used to obtain data on age, sex, diagnosis (GPA or MPA) and laboratory parameters at admission. The glomerular filtration rate (eGFR) was estimated using the Chronic Kidney Disease Epidemiology Collaboration (CKD-EPI) equation^42^.

### Scoring systems

At admission, the Birmingham Vasculitis Activity Score (BVAS) version 3 was calculated as described previously^43^. The BVAS is assessed on a scale of 0 to 63, with a score of 0 indicating the absence of disease activity and higher scores indicating active disease. The Simplified Acute Physiology Score (SAPS) II was calculated according to published guidelines^44^. The Acute Physiology and Chronic Health Evaluation (APACHE) II score was assessed within the first 24 h of the ICU stay, with the most severe result used^45^. To calculate the Sequential Organ Failure Assessment (SOFA) score, the function of six major organ systems (cardiovascular, respiratory, renal, hepatic, CNS, and coagulation) were evaluated^46^.

### Definitions

Requirement of intensive care treatment was defined at admission and calculated by the time between admission to the intensive care unit (ICU) or intermediate care unit (IMC) and relocation to the non-ICU/non-IMC medical ward, all patients required critical care treatment > 24 h. KRT was performed intermittently in all cases. Indications of KRT included severe electrolyte and acid–base abnormalities, volume overload and encephalopathy. KRT was terminated when eGFR surpassed 15 mL/min/1.73 m^2^ and there was no hyperkalemia, heart failure, edema, and encephalopathy.

### Renal histopathology

A renal pathologist evaluated all kidney biopsies being blinded to clinical data and analysis. Within a kidney biopsy, the percentage of glomeruli affected by necrosis, crescents, and global sclerosis was calculated as a fraction of the total number of glomeruli. The fraction of normal glomeruli were categorized within each kidney biopsy (0–25%, 26–50%, 51–75%, or 76–100%) as previously described^47^. In addition, the degree of interstitial fibrosis/tubular atrophy (IF/TA) was quantified. Histopathological subgrouping was performed as previously described by Berden et al. (focal, crescentic, mixed, or sclerotic class), and ARRS according to Brix et al. (low, medium, or high risk)^48,49^.

### Remission induction therapy

Glucocorticoids (GCs) were administered either as intravenous pulse therapy or orally with a tapering schedule. Additional plasma exchange (PEX) was performed in case of severe kidney injury and/or alveolar hemorrhage. Intravenous rituximab (RTX) was administered as four doses at 375 mg/m^2^ every week, and RTX was not administered within 48 h before PEX treatment. Intravenous cyclophosphamide (CYC) was administered adjusted for age and kidney function as three doses up to 15 mg/kg every 2 weeks and every 3 weeks thereafter. Combination therapy was administered as four intravenous doses at 375 mg/m^2^ RTX every week and two intravenous doses at 15 mg/kg CYC every 2 weeks.

### Statistical methods

Normal distribution was tested by using the Shapiro–Wilk test. Non-normally distributed continuous variables are presented as median and interquartile range (IQR), the Mann–Whitney *U* test was used to determine differences in medians. Categorical variables as frequency and percentage, non-parametric between-group-comparisons were performed with Pearson’s Chi-square test. Based on receiver operator curves (ROC) and the area under the curve (AUC), maximized Youden’s index (sensitivity + specificity − 1) to discriminate groups was evaluated. Survival curve comparisons were performed by log rank (Mantel–Cox) testing. For multiple linear regression analyses, covariates were retained to significant differences in between-group-comparisons to avoid model over-fit. Data analyses were performed with GraphPad Prism (version 8.4.3 for MacOS, GraphPad Software, San Diego, California, USA), multiple comparisons were performed using IBM SPSS Statistics (version 27 for MacOS, IBM Corporation, Armonk, NY, USA).

## Data Availability

Deidentified data are available on reasonable request from the corresponding author.

## References

[1] Jennette JC (2013). 2012 revised International Chapel Hill Consensus Conference Nomenclature of Vasculitides. Arthritis Rheumatol..

[2] Hruskova Z (2015). Characteristics and Outcomes of Granulomatosis With Polyangiitis (Wegener) and Microscopic Polyangiitis Requiring Renal Replacement Therapy: Results From the European Renal Association-European Dialysis and Transplant Association Registry. Am. J. Kidney Dis..

[3] Yates M (2016). EULAR/ERA-EDTA recommendations for the management of ANCA-associated vasculitis. Ann. Rheum. Dis..

[4] Specks U (2013). Efficacy of remission-induction regimens for ANCA-associated vasculitis. N. Engl. J. Med..

[5] Stone JH (2010). Rituximab versus cyclophosphamide for ANCA-associated vasculitis. N. Engl. J. Med..

[6] Jones RB (2010). Rituximab versus cyclophosphamide in ANCA-associated renal vasculitis. N. Engl. J. Med..

[7] Hogan SL (2005). Predictors of relapse and treatment resistance in antineutrophil cytoplasmic antibody-associated small-vessel vasculitis. Ann. Intern. Med..

[8] Pagnoux C (2008). Predictors of treatment resistance and relapse in antineutrophil cytoplasmic antibody-associated small-vessel vasculitis: Comparison of two independent cohorts. Arthritis Rheumatol..

[9] Flossmann O (2011). Long-term patient survival in ANCA-associated vasculitis. Ann. Rheum. Dis..

[10] Guillevin L (2011). The Five-Factor Score revisited: Assessment of prognoses of systemic necrotizing vasculitides based on the French Vasculitis Study Group (FVSG) cohort. Medicine (Baltimore).

[11] Cruz BA (2003). Prognosis and outcome of 26 patients with systemic necrotizing vasculitis admitted to the intensive care unit. Rheumatology (Oxford).

[12] Monti S (2015). Life-threatening onset of systemic vasculitis requiring intensive care unit admission: A case series. Clin. Exp. Rheumatol..

[13] Haviv Y (2019). Patients with vasculitides admitted to the intensive care unit: Implications from a single-center retrospective study. J. Intensive Care Med..

[14] Khan SA, Subla MR, Behl D, Specks U, Afessa B (2007). Outcome of patients with small-vessel vasculitis admitted to a medical ICU. Chest.

[15] Demiselle J (2017). Patients with ANCA-associated vasculitis admitted to the intensive care unit with acute vasculitis manifestations: A retrospective and comparative multicentric study. Ann. Intensive Care.

[16] Holguin F, Ramadan B, Gal AA, Roman J (2008). Prognostic factors for hospital mortality and ICU admission in patients with ANCA-related pulmonary vasculitis. Am. J. Med. Sci..

[17] Befort P (2013). Prognosis and ICU outcome of systemic vasculitis. BMC Anesthesiol..

[18] Zhang Y (2021). Predictors of mortality in critically ill patients with antineutrophil cytoplasmic antibody-associated vasculitis. Front. Med. (Lausanne).

[19] Burkhardt O (2007). Predicting outcome and survival in patients with Wegener's granulomatosis treated on the intensive care unit. Scand. J. Rheumatol..

[20] Frausova D, Brejnikova M, Hruskova Z, Rihova Z, Tesar V (2008). Outcome of thirty patients with ANCA-associated renal vasculitis admitted to the intensive care unit. Ren. Fail..

[21] Ge Y (2020). Outcome predictors of biopsy-proven myeloperoxidase-anti-neutrophil cytoplasmic antibody-associated glomerulonephritis. Front. Immunol..

[22] Wacrenier S, Boud'hors C, Piccoli G, Augusto JF, Brilland B (2021). Commentary: Outcome predictors of biopsy-proven myeloperoxidase-anti-neutrophil cytoplasmic antibody-associated glomerulonephritis. Front. Immunol..

[23] Hakroush S (2020). Histopathological findings predict renal recovery in severe ANCA-associated vasculitis requiring intensive care treatment. Front. Med. (Lausanne).

[24] Hakroush S (2021). Systematic histological scoring reveals more prominent interstitial inflammation in myeloperoxidase-ANCA compared to proteinase 3-ANCA glomerulonephritis. J. Clin. Med..

[25] Hakroush S, Tampe D, Korsten P, Strobel P, Tampe B (2021). Bowman's capsule rupture links glomerular damage to tubulointerstitial inflammation in ANCA-associated glomerulonephritis. Clin. Exp. Rheumatol..

[26] Tampe D, Strobel P, Korsten P, Hakroush S, Tampe B (2021). Consideration of therapeutic plasma exchange in association with inflammatory lesions in ANCA-associated glomerulonephritis: A real-world retrospective study from a single center. Front. Immunol..

[27] Tampe D, Korsten P, Strobel P, Hakroush S, Tampe B (2021). Comprehensive analysis of sex differences at disease manifestation in ANCA-associated glomerulonephritis. Front. Immunol..

[28] Tervaert JW (2007). Vasculitis and the intensive care. Acta Clin. Belg..

[29] Wilson A, Yu HT, Goodnough LT, Nissenson AR (2004). Prevalence and outcomes of anemia in rheumatoid arthritis: A systematic review of the literature. Am. J. Med..

[30] Nielsen OH, Ainsworth M, Coskun M, Weiss G (2015). Management of iron-deficiency anemia in inflammatory bowel disease: A systematic review. Medicine (Baltimore).

[31] Bergamaschi G (2010). Prevalence and pathogenesis of anemia in inflammatory bowel disease. Influence of anti-tumor necrosis factor-alpha treatment. Haematologica.

[32] Voulgarelis M (2000). Anaemia in systemic lupus erythematosus: Aetiological profile and the role of erythropoietin. Ann. Rheum. Dis..

[33] Weiss G, Goodnough LT (2005). Anemia of chronic disease. N. Engl. J. Med..

[34] Kawamura T (2017). Anaemia is an essential complication of ANCA-associated renal vasculitis: A single center cohort study. BMC Nephrol..

[35] Culleton BF (2006). Impact of anemia on hospitalization and mortality in older adults. Blood.

[36] Go AS (2006). Hemoglobin level, chronic kidney disease, and the risks of death and hospitalization in adults with chronic heart failure: The Anemia in Chronic Heart Failure: Outcomes and Resource Utilization (ANCHOR) Study. Circulation.

[37] French C (2019). Erythropoietin in critical illness and trauma. Crit. Care Clin..

[38] Mohanram A (2004). Anemia and end-stage renal disease in patients with type 2 diabetes and nephropathy. Kidney Int..

[39] Iseki K, Ikemiya Y, Iseki C, Takishita S (2003). Haematocrit and the risk of developing end-stage renal disease. Nephrol. Dial. Transplant..

[40] Chant C, Wilson G, Friedrich JO (2006). Anemia, transfusion, and phlebotomy practices in critically ill patients with prolonged ICU length of stay: A cohort study. Crit. Care.

[41] Tanner L (2022). Influence of anaemia in severely injured patients on mortality, transfusion and length of stay: An analysis of the TraumaRegister DGU((R)). Eur. J. Trauma Emerg. Surg..

[42] Levey AS (2009). A new equation to estimate glomerular filtration rate. Ann. Intern. Med..

[43] Mukhtyar C (2009). Modification and validation of the Birmingham Vasculitis Activity Score (version 3). Ann. Rheum. Dis..

[44] Le Gall JR, Lemeshow S, Saulnier F (1993). A new Simplified Acute Physiology Score (SAPS II) based on a European/North American multicenter study. JAMA.

[45] Knaus WA, Draper EA, Wagner DP, Zimmerman JE (1985). APACHE II: A severity of disease classification system. Crit. Care Med..

[46] Singer M (2016). The Third International Consensus Definitions for Sepsis and Septic Shock (Sepsis-3). JAMA.

[47] Hilhorst M (2013). Estimating renal survival using the ANCA-associated GN classification. J. Am. Soc. Nephrol..

[48] Berden AE (2010). Histopathologic classification of ANCA-associated glomerulonephritis. J. Am. Soc. Nephrol..

[49] Brix SR (2018). Development and validation of a renal risk score in ANCA-associated glomerulonephritis. Kidney Int..

